# Behavioral indicators of heterogeneous subjective experience in animals across the phylogenetic spectrum: Implications for comparative animal phenomenology

**DOI:** 10.1016/j.heliyon.2024.e28421

**Published:** 2024-03-24

**Authors:** Louis N. Irwin

**Affiliations:** Department of Biological Sciences, University of Texas at El Paso, El Paso, TX, USA

**Keywords:** Animal cognition, Experiential profile, Behavior, Ecology, Ethology, Phylogeny, Consciousness

## Abstract

This behavioral study was undertaken to provide empirical evidence in favor of or opposed to the notion that animals across a wide breadth of the animal kingdom have subjective (personal) experience that varies with their lifestyles, ecological constraints, or phylogeny. Twelve species representing two invertebrate phyla and six vertebrate classes were observed unobtrusively in 15-min episodes, during which three modes of behavior (volitional, interactive, and egocentric) were quantified according to the frequency, variety, and dynamism of each mode. Volitional behavior was the most prevalent and dynamic mode for nearly all species, largely without regard to phylogenetic position. Interactive behavior likewise varied inconsistently across the entire evolutionary spectrum. Egocentric behavior was concentrated among the avian and mammalian species, but evidence of it were observed in the invertebrate species as well. Diagrams of the matrix constructed from the three qualitative modes and three quantitative attributes for each mode provide a metaphorical representation of the unique experiential profile of each species. To the extent that these behavioral measures correlate with the nature of the animal's subjective experience, they support the growing view that phenomenology is heterogeneous, multimodal, and non-linear in extent across the animal kingdom.

## Highlights

• Three behavioral indicators of subjective experience were quantified in 12 species.
• Phenomenology correlated more strongly with life style than habitat or phylogeny.
• Heat diagrams provide metaphors for species-specific phenomenological diversity.
• Phenomenology is multimodal and heterogeneous across the animal kingdom.

## Graphical abstract

**Figure:**
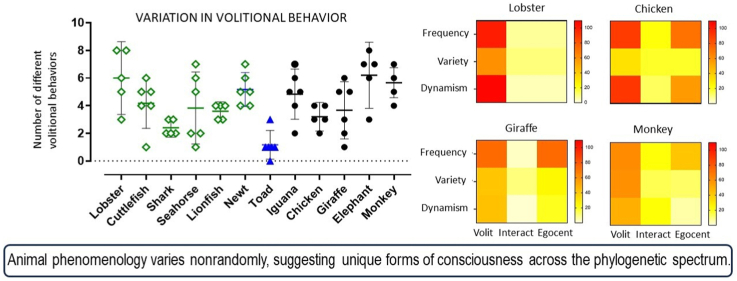

## 1 Introduction

Few neurobiologists today retain the Cartesian notion that consciousness is solely a human experience. Increasingly, cognitive scientists and students of behavior and the nervous system are coming to the view that phenomenological awareness has deep evolutionary roots and is widespread across the animal kingdom. The argument for this view has been advanced most compellingly by Feinberg and Mallatt [[1], [2], [3]], with increasing numbers of scientists in agreement [[4], [5], [6], [7], [8]].

Yet the Cartesian legacy in favor of human phenomenology to the exclusion of a greater or lesser portion of the animal kingdom that varies from one theorist to another persists in the face of overwhelming evidence that most if not all animals with a sufficiently complex nervous system are sentient to some degree [[9], [10], [11], [12], [13], [14]]. This likely reflects the fact that phenomenal experience in humans is considered self-evident since others can share through language with subtlety and detail mental states that are comparable to our own subjective experience [15,16]. Logic dictates that whatever function subjective awareness serves for humans must do likewise to some degree and in some fashion in all species that actively engage with their environments. That being the case, the scientific question becomes, What is the nature of that phenomenological experience in other animals and how might it be assessed?

While direct access to subjective experience is inaccessible by an outside observer [17,18], indirect access through behavioral indicators in humans, validated by verbal testimony, can suggest behaviors in animals that could serve as proxies for subjective experience [[19], [20], [21]]. Based on that rationale, three modes of behavior were selected in this study as proxies for different cognitive processes that in humans are clearly associated with different aspects of subjective experience. By quantifying each mode in three different ways, a matrix of experiential profiles was generated for each species. This information was then used to test the competing hypotheses that the ecology, phylogenetic position, or lifestyle best explains the nature of the animal's presumed subjective experience.

Another barrier to insight into the nature of subjective experience in animals has been the lack of an appropriate vocabulary [[22], [23], [24]] or way of envisioning heterogeneous and graded degrees of phenomenological experience [25]. I agree with Ginzburg and Jablonka [11] that the term ‘consciousness’ carries such historical baggage and ambiguity of meaning that ‘subjective experience’ is preferable. Therefore, the topic of this paper is defined as the subjective (personal) awareness of an animal's phenomenological experience [7,26,27].

One strategy recently gaining favor is the creation of conceptual profiles for visualizing the variable complexity of subjective experience characteristic for different species [25,28,29]. This paper proposes a unique visual metaphor for subjective experience that reveals a weak tendency for increased complexity of experiential profiles in the amniotic vertebrates studied, but with profiles for representatives from most taxa that were not simple.

## 2 Materials and methods

### 2.1 Subjects

This was an observational study of 12 animal species, with representatives from two invertebrate phyla (Arthropoda and Mollusca) and all six extant vertebrate classes (Chondrichthyes, Osteichthyes, Amphibia, Reptilia, Aves, and Mammalia). Animals were chosen for observation with no set criteria other than phylogenetic diversity and the observer's ability to view all the selected aspects of their behavior clearly but unobtrusively. Five species were marine (lobster, cuttlefish, shark, seahorse, and lionfish), two were riparian (newt and toad), and five were terrestrial (iguana, chicken, elephant, giraffe, and colobus monkey). While the two riparian species reproduce in fresh water, the newt lives predominantly in water so is classified with the marine species as aquatic, and the adult toad lives in a terrestrial habitat but differs in nearly all aspects from the other terrestrial species, so is listed as a riparian representative. Table 1 gives a complete list of species with their scientific names, sex where ascertainable, and locations of observation.

**Table 1:** Species, taxonomy, and location of observations.

Animal *– Species*	Taxonomy	Place observed
Caribbean Spiny Lobster – *Panulirus argus*	Arthropod/Crustacean	Denver Aquarium
Cuttlefish – *Sepia* sp.	Mollusk/Cephalopod	Denver Aquarium
Brown Shark – *Carcharinus plumbeus*	Vertebrate/Cartilaginous fish	Denver Aquarium
Seahorse – *Hippocampus* sp.	Vertebrate/Bony fish	Denver Aquarium
Lionfish - *Pterois volitans*	Vertebrate/Bony fish	Denver Aquarium
Eastern Newt – *Notophthalmus viridescens*	Vertebrate/Amphibian/Urodele	Denver Zoo
Colorado River Toad - *Incilius alvarius*	Vertebrate/Amphibian/Anuran	Denver Aquarium
Rhinocerus Iguana (F) - *Cyclura ornate*	Vertebrate/Reptile/Squamata	Denver Zoo
Chicken (M) – *Gallus gallus*	Vertebrate/Aves/Galliformes	Kauai, Hawaii^a^
Asian Elephant (M)– *Elephas maximus*	Vertebrate/Mammal/Proboscidea	Denver Zoo
Reticulated Giraffe (F) – *Giraffa camelopardolis*	Vertebrate/Mammal/Artiodactyl	Denver Zoo
Black and White Colobus (M) – *Colobus guereza*	Vertebrate/Mammal/Primate	Denver Zoo

a Open range When the sex of all subjects observed for a given species was the same, it is indicated by F for females and M for males in parentheses. The sex of subjects in all other species was not ascertainable.

### 2.2 Behavioral observations

Individual animals were scored by the same single observer at the zoo (around midday) or aquarium (late afternoon) in Denver, Colorado, U.S.A., or in an open field of chickens on Kauai, Hawaii (mid-morning). Having all data collected by the same observer had the virtue of eliminating inter-observer variation, but introduced the possibility of bias held by the sole observer. To mitigate the latter, trial observations of all species were made to ensure that the designated criteria for distinguishing the different modes of behavior and their quantitative assessments could be applied consistently across all species. All observations were 15 min in duration, and were unobtrusive with no evident influence on the behavior of the subjects. The distance between observer and subject was kept at the maximum freasible for accurate scoring. The goal was to observe enough epidsodes for coefficients of variation (CV) in the frequency of the most common activity to be lower than 40%. This was achieved with either 5 or 6 separate observational episodes for all species except the toad (CV = 91%), which had a very low baseline level of activity, giraffe (CV = 56%), and cuttlefish (CV = 64%). Different individuals were scored for each observational episode whenever possible.

The goal of this study was to quantify experiential profiles through proxy behaviors that in humans are associated with subjective experience, meaning types of behavior associated with different aspects, facets, or reflections of consciousness in humans, and presumably in non-human animals to variable species-specific degrees. Three different modes of behavior assessed for this purpose were volitional, interactive, and egocentric. Each mode was quantitatively measured in three ways, according to its frequency, variety, and dynamism. This follows the strategy advocated by Seth [30] and Pennartz [25] for assessing consciousness through analysis across multiple properties, preferably with a combination of qualitative and quantitative elements.

#### 2.2.1 Qualitative modes of behavior

**Volitional** behavior consisted of kinetic actions that appeared to be deliberative, intentional, and goal-directed. Generally, any movement of the subject from one place to another, or initiation of behavior that altered the subject's condition or environment, was considered to be a unit of volitional behavior, provided the motion was clearly not a reflexive reaction to sudden stimuli. Examples are shown in the Supplemental Videos (SV) by the deliberative walk of the lobster and different actions by the iguana (SV1); by the monkeys that deliberately move apart after their brief interaction (SV2); by the elephants that walk away in opposite directions, followed by the dive of one of them into the water (SV3), and by the giraffe's deliberate walk to a new location (SV4).

Neurobiological mechanisms related to volitional behavior include activity in the anterior cingulate cortex and medial prefrontal cortex in humans associated with awareness of intentional actions [31], organization in vertebrates of an upper brain stem system for conscious action control [32], and a number of studies on the cortical basis of conscious volition down to the single-neuron level [33]. The value of subjective awareness for volitional motility has previously been pointed out [25,34].

**Interactive** behavior was defined as any contact, communication, aggression toward, defense against, engagement with, or reaction to a conspecific or allospecific individual. It included reaction to exteroceptive stimulation, like sounds or actions at a distance made by other individuals of the same or different species. Examples of interactive behavior are seen at the start of the sequence with monkeys in SV2 and elephants in SV3.

Interaction with other organisms typically requires subjective awareness [35], as does analysis of external signals from other animals or the environment that bear on the animal's survival [36]. So all the usual neurobiological mechanisms of perception ― and in social species, social signaling and reaction ― are clearly tied to interactive behavior.

**Egocentric** behavior entailed somatic attention to or awareness of the animal's own body. Examples included scratching, yawning, self-grooming, washing, eating, and intentional reactions to stimulation from the environment that impinged directly on the animal's body. Not all such actions necessarily require subjective awareness, so only actions in alert animals that appeared to be initiated spontaneously in a non-reflexive manner were scored in this category. An example of egocentric behavior was eating followed by smacking of the lips in the giraffe (SV4).

Egocentric behavior entails self-directed attention, which involves central and peripheral nervous system mechanisms for mapping the body, the environment, and the relationship between the organism and its environment [37,38]. It is presumed to imply a sense of self and often reflect the animal's affective state [21], for which neurochemically specific circuits in the limbic system of vertebrates are well-known, and a variety of invertebrate neurobiological correlates are now under study [39].

#### 2.2.2 Quantitative attributes of each mode of behavior

Each mode of behavior was assessed for the extent to which three different attributes were observed during each observational period.

*Frequency* was a measure of the degree to which a given mode of behavior was exhibited during the period of observation. The number of 60-s intervals during which the subject displayed volitional, interactive, and/or egocentric behavior was noted, and reported as a percentage of the total number of 60-s intervals composing each observational episode.

*Variety* provided an indication of the range and heterogeneity of a subject's behavioral repertoire. The variety of volitional behaviors was measured by the number of new and distinguishable deliberative actions taken. Interactive behavior was quantified by the number of new and different encounters with or reaction to another individual of the same or different species. Egocentric variation was quantified by the number of different forms of self-directed activity that were displayed during the observational period.

*Dynamism* was a measure of how often a new behavior within each mode was initiated, or frequency with which a change in behavior occurred. Each mode of behavior could be constant in form for the duration of an observational period, or it could change frequently from one form to another. The number of times that a new form of volitional, interactive, or egocentric behavior was displayed provided a quantitative measure of how dynamically each mode of behavior was exhibited.

#### 2.2.3 Representation of behavioral profiles

The three modes of behavior, each quantified by three different metrics, provided the opportunity to express a unique two-dimensional profile of behavior for each species. The sum of data points in each modal-attribution category for each species were recorded for volitional and egocentric behaviors within each 15-min observation, while the sum of interactions were extrapolated to a 60-min period (to equalize the order of magnitude of interactions with the other two modes for graphic clarity). The resulting behavioral profiles were expressed as 3 × 3 heat diagrams for each species, coded for intensity by the measure of each quantitative attribute for each behavioral mode.

### 2.3 Statistical analysis

Most animals show great variation in quantitative measures of behavior, related to time of day or night, physical features of the environment, physiological and affective state, social hierarchies or imperatives, and other factors. No single observational episode can be viewed necessarily as “typical; ” each episode is a unique sample of the animal's experience in that instance. For this reason, data are shown as individual values from each observational episode, with no data points discarded. All error bars show confidence intervals (CI) of 95% across all observational episodes for each species. Though the sample sizes (n = 5–6) are relatively small the CI of 95% provides an objective measure of the degree of variation in each metric chosen for study.

The probability that quantitative measures of behavior differed by more than chance across multiple species was assessed by the non-parametric Kruskal-Wallace (KW) 1-way analysis of variance (ANOVA). A non-parametric test was chosen because of the limited sample sizes and the lack of a normal distribution (symmetrical spread of measures on either side of a central mean) in most of the data. Non-parametric statistics test for propbability based on degree of data overlap rather than how spread out the values are. When groups of species collectively were compared with each other, as when aquatic and terrestrial species were contrasted, a nested 1-way ANOVA with Tukey's correction for multiple comparisons was applied. This provides a test for how overall trends within a group of subjects compare with overall trends in another group.

To test the probability that behavioral profiles varied as a function of evolutionary age, the mean quantitative measures of each behavioral parameter were plotted against phylogenetic distance, as measured by age of divergence from the last common ancestor between each species and humans, based on data from Kumar and Hedges [40]. The probability that the slope of this linear regression differed from zero (indicating an evolutionary trend over time) was then computed.

Fig. 7 shows behavioral profiles as 3 × 3 heat diagrams with the quantitative measure in rows and the qualitative mode of behavior in columns for each species. The probability that the underlying data differed significantly for each species in comparison with the data matrix for the lobster (the most ancient species) was tested by the Chi Square statistic. The test was then repeated using data from the monkey (the most recently evolved species) for the expected values. Finally, pairwise comparisons of selected species that appeared similar were tested. Not all comparisons could be made because of the presence of some zero data points, which invalidate the Chi Square test. The results of all Chi Square tests are shown in Appendix A.

All data analysis, statistical evaluation, and graphing was carried out with GraphPad Prism®, version 9.4.1. The null hypothesis probabilities―whether exact, approximate, or less than―are shown just as reported by the software.

## 3 Results

### 3.1 Volition

**Fig. 1:**
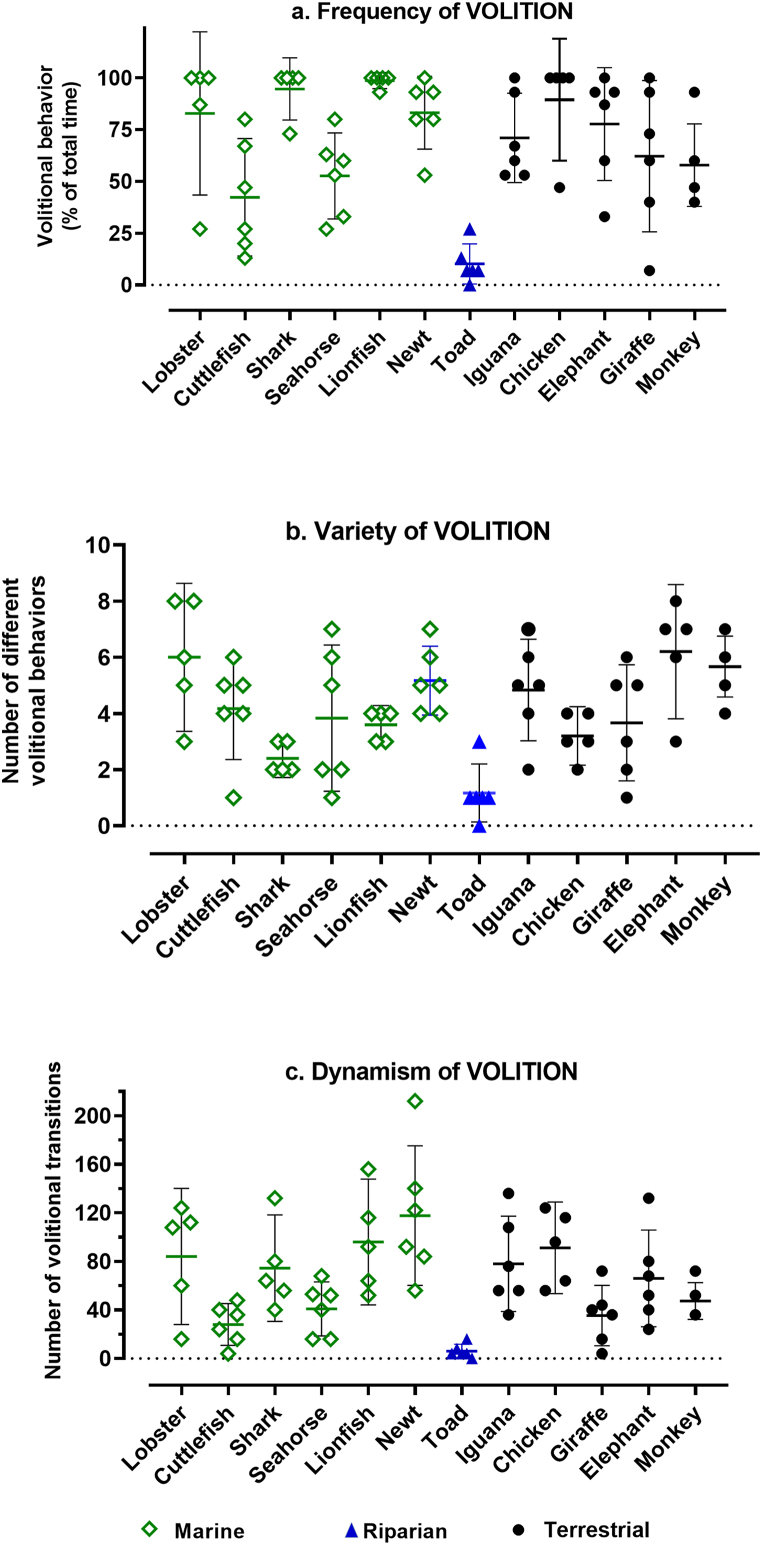
**Frequency, Variety, and Dynamism of Volitional Behavior**. Each data point was obtained during a 15-min observational episode, and reported per 15 min (a and b), or extrapolated to 60 min (c). Error bars show CI = 95% and horizontal lines mark the mean for each dataset. Evolutionary distance decreases roughly from left to right. Interspecies differences overall were significantly non-random for (a) *frequency* (P∼0.0001), (b) *variety* (P∼0.0006), and (c) *dynamism* (P < 0.0001).

The *frequency* of volitional activity was substantial in all species, except for the typically sedentary toad (Fig. 1a). Despite large variation in the volitional activity of some species over observational episodes, the frequency distribution of volitional activity across species overall varied significantly (KW = 38.45, n = 12 groups, P∼0.0001), as did the frequency of volitional activity across aquatic species only (KW = 19.75, n = 6 groups, P∼0.0014). However, a nested 1-way ANOVA with Tukey's correction for multiple comparisons showed that volitional frequency of aquatic species as a group did not differ significantly from that of terrestrial species as a group (q = 0.672, df = 65, P = 0.883), although the toad displayed significantly less volitional activity compared to either the aquatic (q = 7.682, df = 65, P < 0.0001) or terrestrial (q = 7.220, df = 65, P < 0.0001) subgroup of animals.

The *variety* of volitional behaviors (Fig. 1b) across all species likewise differed significantly (KW = 32.76, n = 12 groups, P∼0.0006). Differences were also significant among both the aquatic species only (KW = 11.82, n = 6 groups, P∼0.037) and the terrestrial species only (KW = 10.58, n = 5 groups, P∼0.032). The nested 1-way ANOVA showed that the variety of volitional activity of aquatic species as a group did not differ significantly from that of terrestrial species as a group (q = 1.58, df = 65, P = 0.506), but the toad displayed significantly less volitional variety compared to either the aquatic (q = 5.397, df = 65, P = 0.0009) or terrestrial (q = 6.239, df = 69, P = 0.0001) subgroup of animals.

The *dynamism* of volitional behaviors (Fig. 1c) across all species also differed significantly (KW = 39.95, n = 12 groups, P < 0.0001). And it differed significantly within both the aquatic species only (KW = 17.31, n = 6 groups, P∼0.004) and the terrestrial species only (KW = 10.22, n = 5 groups, P∼0.037). The nested 1-way ANOVA showed that the dynamism of volitional activity of aquatic species as a group did not differ significantly from that of terrestrial species as a group (q = 1.32, df = 65, P = 0.621), but the toad displayed significantly less volitional dynamism compared to either the aquatic (q = 5.263, df = 65, P = 0.0012) or terrestrial (q = 4.47, df = 65, P = 0.0068) subgroup of animals.

**Fig. 2:**
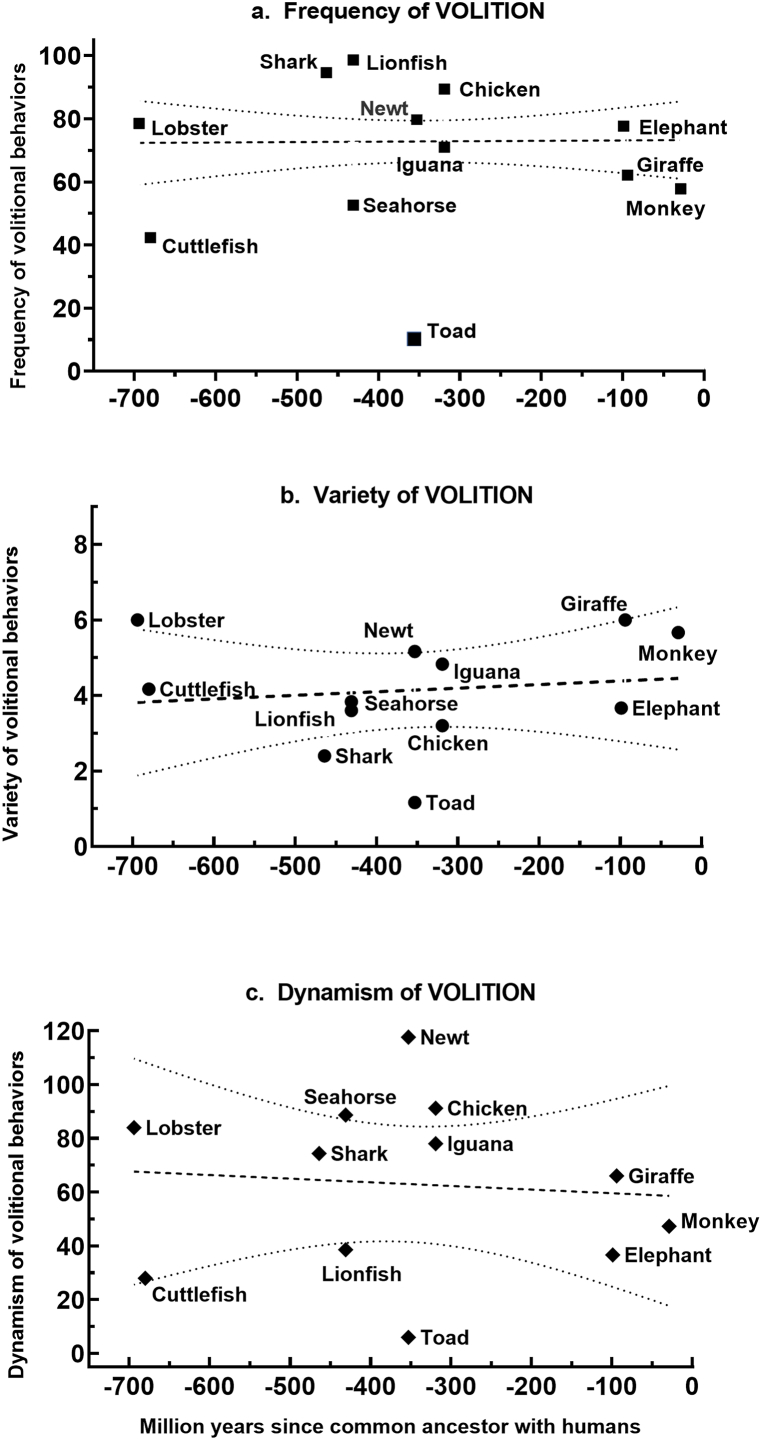
**Linear regression of frequency, variety, and dynamism of volitional behavior against evolutionary distance.** The mean value of attributes from all observational episodes (n = 5–6) for each species were plotted against evolutionary distance to generate the best fitting line (bold dashes) with 95% confidence bands (light dashes) for its slope. The slopes did not differ from zero for (a) *frequency* (slope = 0.0013, F = 0.0064, P = 0.937), (b) *variety* (slope = 0.0022, F = 0.187, P = 0.675), or (c) *dynamism* (slope = −0.0136, F = 0.080, P = 0.783).

In summary, the *frequency*, *variety*, and *dynamism* of **volitional** behavior varied significantly across the dataset as a whole, and usually within habitat categories, tending to be species-specific and not correlated with broad habitat categories. An alternative to the ecological explanation for behavioral patterns is the hypothesis that they vary with phylogeny. This was tested by examining the linear regression of the quantitative attributes of volitional behavior with evolutionary distance from humans. Fig. 2a–c reveals that all three attributes of volitional behavior bore no quantitative relationship to evolutionary distance, as measured by age of divergence from the last common ancestor with humans.

### 3.2 Interaction

**Fig. 3:**
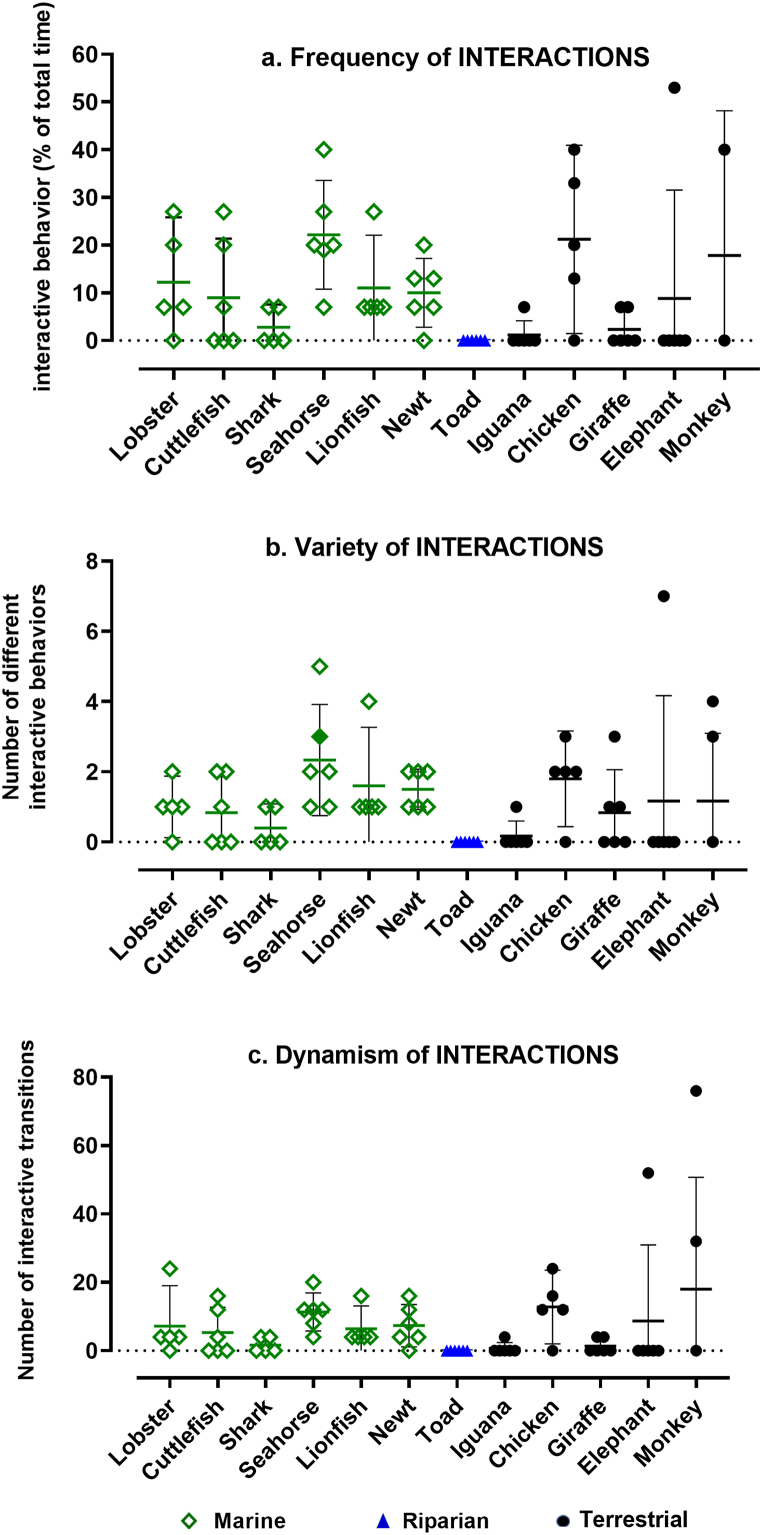
**Frequency, variety, and dynamism of interactive behavior.** Each data point was obtained during a 15-min observational episode, and reported per 15 min (a and b), or extrapolated to 60 min (c). Error bars show CI = 95% and horizontal lines mark the mean for each dataset. Interspecies differences overall were significantly non-random for (a) *frequency* (P*∼*0.0043), (b) *variety* (P*∼*0.0049), and (c) *dynamism* (P*∼*0.0055).

For most of the studied species, the *frequency* of **interactive** behavior was quite variable, with the toad again providing a stark exception (Fig. 3a). This would be expected due to the opportunistic nature of conspecific and interspecific contacts. The lack of interaction among the elephants during all but one observational episode (Fig. 3a), was probably an artifact of their confinement in a zoo, as they are highly social animals in their natural habitats. Overall, the frequency of interactive behavior varied significantly across all species studied (KW = 27.18, *n* = 12 groups, *P∼*0.0043). However, differences in the frequency of interactions was not quite significant among the aquatic species (KW = 9.78, *n* = 12 groups, *P∼*0.082) and not significant among the terrestrial species (KW = 6.23, *n* = 12 groups, *P∼*0.183). A pairwise comparison by 1-way ANOVA for an overall difference in frequency of interaction between aquatic vs terrestrial species was not significant: (q = 0.601, df = 65, *P* = 0.905).

The *variety* of **interactive** behavior (Fig. 3b) also varied significantly across all species (KW = 26.79, *n* = 12 groups, *P∼*0.0049). Differences in the variety of different interactions was marginally significant among the aquatic species (KW = 10.90, *n* = 6 groups, *P∼*0.0533) and not significant among the terrestrial species (KW = 5.236, *n* = 5 groups, *P∼*0.255). A pairwise comparison by 1way ANOVA for an overall difference in variety of interaction between aquatic vs terrestrial species was not significant: (q = 1.245, df = 65, *P* = 0.655).

The *dynamism* of **interactive** behavior (Fig. 3c) likewise varied significantly across all species (KW = 26.49, *n* = 12 groups, *P∼*0.0055). However, differences in the number of transitions in interactive behavior were not significant among either the aquatic (KW = 8.907, *n* = 6 groups, *P∼*0.113) or terrestrial (KW = 6.121, *n* = 5 groups, *P∼*0.190) species; and again, the pairwise comparison by 1-way ANOVA for an overall difference in dynamism of interaction between aquatic vs terrestrial species was not significant (q = 0.630, df = 15, *P* = 0.897).

**Fig. 4:**
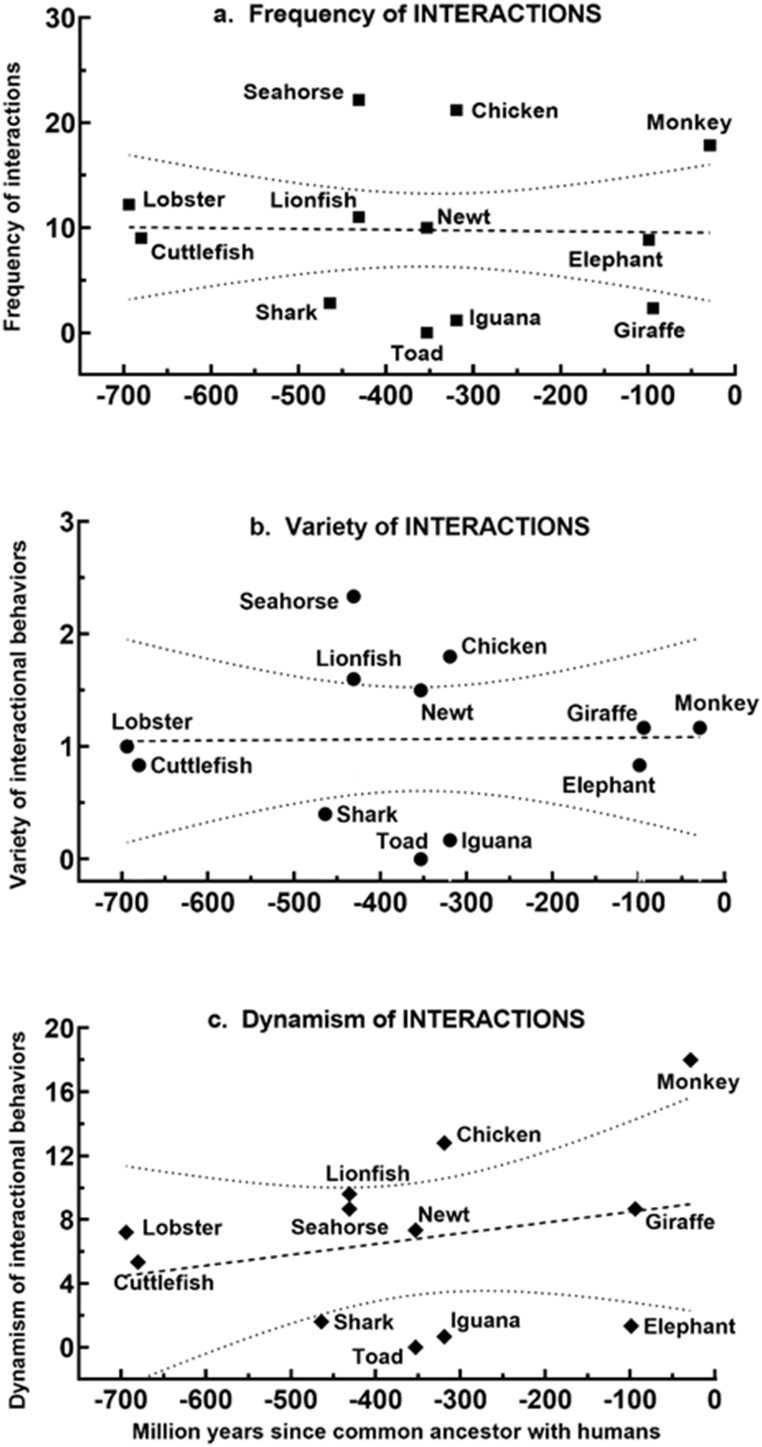
**Linear regression of frequency, variety, and dynamism of interactive behavior against evolutionary distance.** The mean value of attributes from all observational episodes (*n* = 5–6) for each species were plotted against evolutionary distance to generate the best fitting line (bold dashes) with 95% confidence bands (light dashes) for its slope. The slopes did not differ from zero for (a) *frequency* (slope = −0.0008, F = 0.0082, *P* = 0.928), (b) *variety* (slope = 0.0010, F = 0.0027, *P* = 0.960), or (c) *dynamism* (slope = 0.0067, F = 0.741, *P* = 0.410).

In summary, the heterogeneity of **interactive** behavioral profiles varied significantly across species overall for all three quantitative attributes. However, within habitat categories, differences were less evident than with volitional behavior. The possibility that these patterns were attributed to phylogeny was tested by examining the linear regression of the quantitative attributes of interactive behavior with evolutionary distance from humans. Fig. 4a–c reveals that all three attributes of integrative behavior bore no quantitative relationship to evolutionary distance, as measured by age of divergence from the last common ancestor between each species and humans.

### 3.3 Egocentricity

**Fig. 5:**
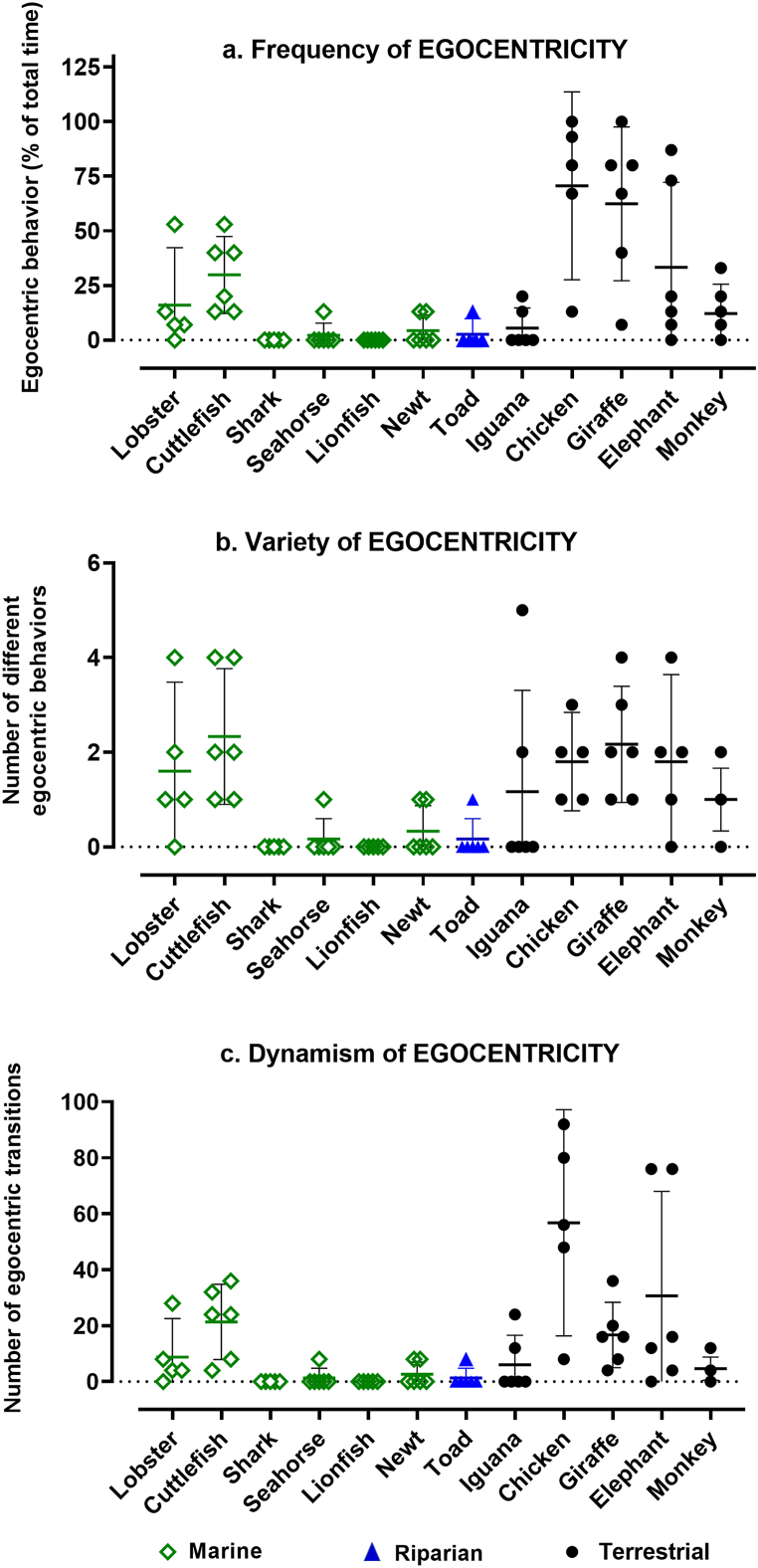
**Frequency, variety, and dynamism of egocentric behavior.** Each data point was obtained during a 15-min observational episode, and reported per 15 min (a and b), or extrapolated to 60 min (c). Error bars show CI = 95% and horizontal lines mark the mean for each dataset. Interspecies differences overall were significantly non-random (P < 00.0001) for all three attributes (*frequency, variety*, and *dynamism).*

The scatter plots for *frequency* of **egocentric** (self-directed) behavior (Fig. 5a) reveal obvious differences across the 12 species, which are statistically significant (KW = 42.90, *n* = 12 groups, *P* < 0.0001). However, similarities are seen both within and across habitat categories. It approached negligible levels for active species, like the shark and lionfish, and for the toad, a primarily immobile sit-and-wait predator. Its apparent absence in these species could be an underestimate, due to the observer's inability to recognize the subtle nature of egocentric activity in those species. Two other marine species ― the lobster and cuttlefish ― have a frequency pattern similar to that of the chicken and mammalian species. Egocentric frequency is noticeably higher in the avian and mammalian species, but differ significantly within that subgroup (KW = 8.12, *n* = 4 groups, *P∼*0.0437).

The same patterns are seen in the plot of the *variety* of egocentric behaviors (Fig. 5b). Differences are obvious across the full range of species, and are statistically significant (KW = 38.04, *n* = 12 groups, P < 0.0001). Again, however, clusters of similar patterns cut across habitat categories, with the lobster and cuttlefish but not other aquatic species resembling the pattern for the terrestrial species.

The same patterns are again seen in the plot of the *dynamism* of egocentric behaviors (Fig. 5c). Differences are obvious across all species as a group, and are statistically significant (KW = 41.48, *n* = 12 groups, *P* < 0.0001). Once more, clusters of similar patterns cut across habitat categories, with the lobster and cuttlefish but not other aquatic species showing a pattern of dynamism similar to that of the terrestrial species, among which patterns of dynamic egocentric behaviors differ significantly (KW = 12.25, *n* = 5 groups, *P∼*0.0156).

**Fig. 6:**
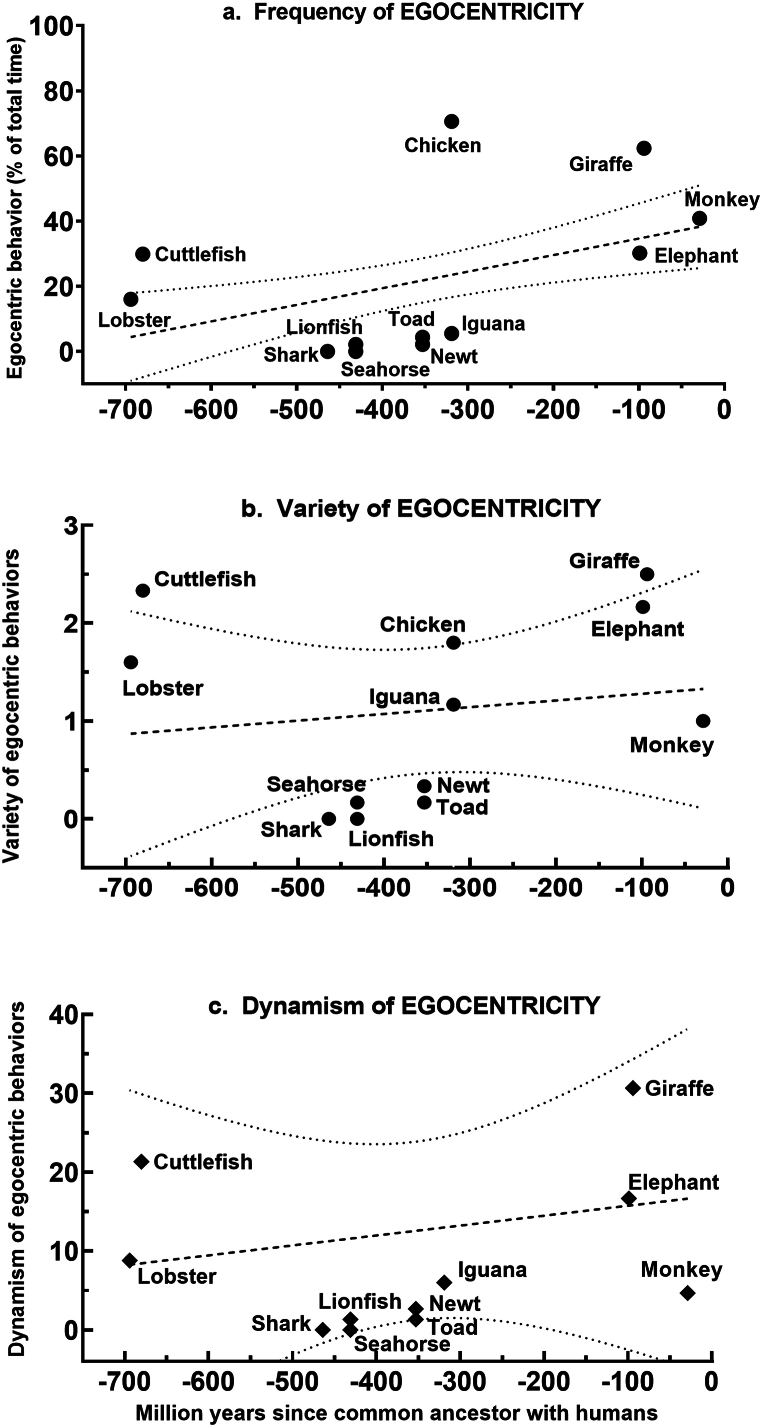
**Linear regression of frequency, variety, and dynamism of egocentric behavior against evolutionary distance**. The mean value of attributes from all observational episodes (n = 5–6) for each species were plotted against evolutionary distance to generate the best fitting line (bold dashes) with 95% confidence bands (light dashes) for its slope. The slope for (a) *frequency* differed significantly (slope = 0.0509, F = 9.13, P = 0.0036) from zero, but not for (b) *variety* (slope = 0.0014, F = 0.232, P = 0.64) or (c) *dynamism* (slope = 0.0216, F = 0.249, P = 0.63).

In summary, the heterogeneity of **egocentric** behavioral profiles varied significantly across species overall for all three quantitative attributes, with clusters of similar patterns cutting across habitat categories. In testing whether any aspect of egocentric behavior could be related to phylogeny, the regression of *frequency* of egocentricity against evolutionary distance (Fig. 6a) revealed a slight positive slope that was statistically significant (slope = 0.051, F = 9.13, *P* = 0.0036), obviously due to the more dynamic egocentricity of the terrestrial species. Similar regressions were not significant for either *variety* (Fig. 6b, slope = 0.0014, F = 0.232, *P* = 0.640) or *dynamism* (Fig. 6c, slope = 0.126, F = 0.249, *P* = 0.628).

**Fig. 7:**
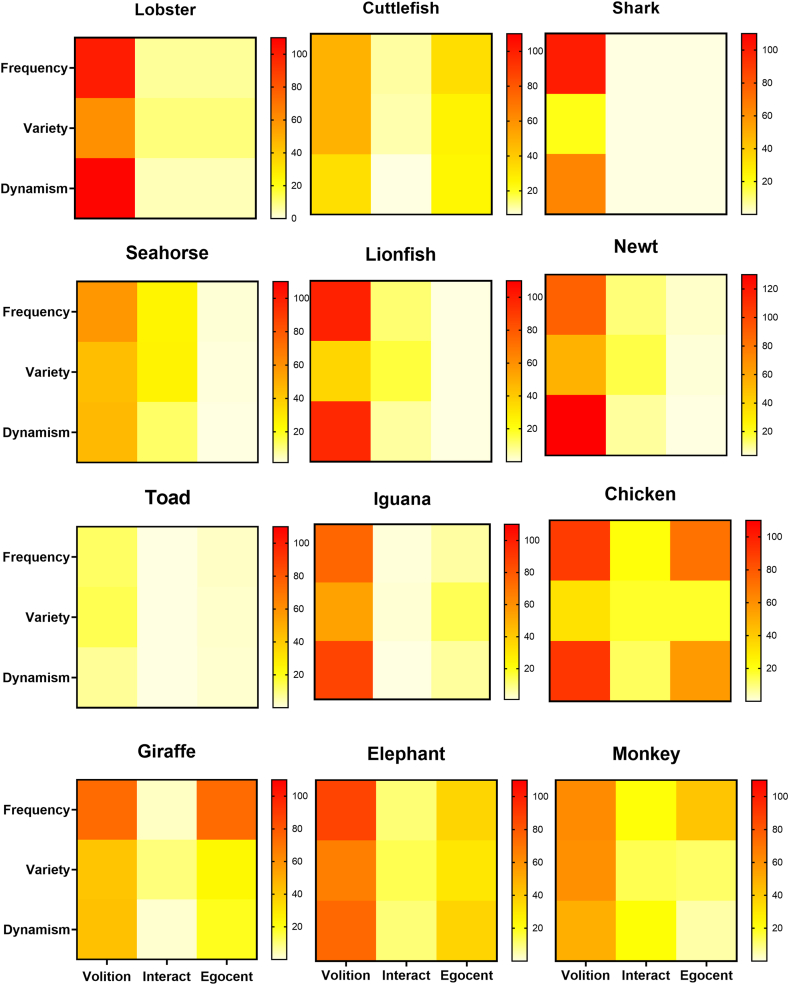
**Behavioral Profiles,** expressed as a mosaic of the sum of quantitative attributes (in rows) for each qualitative behavioral mode (in columns). The value of each qualitative x quantitative square in the matrix is color coded for the sum (darker => higher) of the top 5 total occurrences across all observations for each species. Pair-wise Chi square tests revealed that no two species shared statistically indistinguishable patterns (see Appendix A for probability values), with the possible exception of the lionfish and newt, which are very similar, but could not be evaluated because of zero values in the datasets.

### 3.4 Behavioral profiles

Behavioral profiles in the form of 3 × 3 heat diagrams, coded for the magnitude of each quantitative behavioral measure in the matrix, are shown in Fig. 7. Species are shown in approximate descending order of evolutionary distance from primates. Heterogeneity in behavioral profiles is seen across the entire evolutionary spectrum. All Chi Square tests that were valid (having no zero values in compared data sets) show a significant difference between the behavioral profiles of every species in comparison to the profiles for either the lobster or monkey (Appendix A). However, a tendency for more behavioral complexity in the avian and mammalian taxa than in the phylogenetically older animals is apparent.

## 4 Discussion

That lifestyle provides a better explanation for behavioral profiles is suggested by several specific examples. (1) Active but distantly related species like the lobster, shark, lionfish, and chicken, show prevalent (Fig. 1a) and relatively dynamic (Fig. 1c) **volitional** behavior congruent with their highly mobile lifestyles. (2) The two closely related amphibians, the toad and newt, differ considerably in their *frequency, variety*, and *dynamism* of **volitional** behavior (Fig. 1), consistent with the active foraging of the newt compared to the sit-and-wait strategy of the toad. (3) The three species with the highest mean *frequency* of **interactive** behavior ― seahorse, chicken and monkey ― come from three different taxonomic classes. (4) The *diversity* of **volitional** behavior (Fig. 1b) varied significantly within the aquatic species, on the one hand, and the terrestrial species on the other; and the exceptionally sedentary behavior of the toad featured significantly less variety than that of either the aquatic or terrestrial species.

There does appear to be a trend within the vertebrates toward greater *frequency*, *variety*, and *dynamism* in **egocentricity** among the phylogenetically younger avian and mammalian species (Fig. 5), and the linear regression of *frequency* of **egocentric** behavior against evolutionary age does show a small but significant slope (Fig. 6a). A conservative inference from these observations would be that a cognitive sense of self has grown in complexity with the evolutionary appearance of birds and mammals. However, **egocentric** behavior is prevalent, varied, and dynamic in the two invertebrate species, at opposite ends of the evolutionary spectrum from the vertebrates, so further generalization about evolutionary tendencies in experiential profiles will require much larger sampling across the animal kingdom. y the late nineteenth century, Darwin [41] and Romanes [42], were viewing mental faculties as an emergent consequence of the evolutionary process, graded in complexity and forged by natural selection. As this has become a more prevalent view [[43], [44], [45], [46]], and cognitive ecology has become more influential [29,38,[47], [48], [49], [50]], the likelihood that phenomenology takes different forms in animals with different lifestyles in different ecological settings has gained favor [51]. That it lacks a clear-cut onset, is a multimodal process rather than a bimodal property, and is graded in complexity, is more controversial but is gaining adherents [1,2,7,25,29]. This study provides empirical data consistent with the view that phenomenology is graded in complexity and that consciousness is not an all-or-none phenomenon. The limited number of species in this study is not sufficient to indicate anything about the time of its evolutionary onset, other than that significant cognitive complexity is apparent in animals from clades that evolved as early as the Cambrian.

Another objective of this paper was to suggest a way to conceptualize differences in the phenomenological experience of different species. The heat diagrams in Fig. 7 are consistent with the view that there is no single scale along which experiential complexity varies [28,29]; rather, that multiple patterns of cognition geared toward the demands of species-specific lifestyles have diversified through evolutionary history. For example, the toad and iguana (third row, Fig. 7) are notable for their lack of **egocentric** and **interactive** behavior, consistent with their largely sedentary lifestyles, while the cuttlefish and seahorse, distantly related from amphibians and reptiles, and from each other, show similar profiles to one another consistent with their marine habitats and more active lifestyles. The images in Fig. 7 further suggest that a sit-and-wait predator like the desert toad may have a much simpler mental life than that of the perpetually active chicken, but also that the spiny lobster, representing a group with origins early in the history of animal evolution, may already be capable of relatively complex phenomenological experience.

Using a similar strategy, Birch et al. [28] proposed that consciousness in any species could be represented as a polygon formed by connecting six points corresponding to different aspects of mental experience, each differing in magnitude in a species-specific manner. While the visual profiles that I propose are based on observable behavior rather than assumed phenomenology, the effect is the same: to emphasize that each species has its own distinctive consciousness profile. This includes, of course, organisms lacking consciousness by virtue of a neural capacity that is insufficiently complex.

A major strength of this study lies in the number of species sampled. Nonetheless, the inferences drawn from this study require careful qualification. While larger than most comparative studies of animal cognition, the roster of species observed in this study is far too limited for definitive conclusions about the full scope of behavioral complexity across the entire phylogenetic spectrum. For example, the lack of a clear correlation between behavioral complexity and evolutionary age in this study should not be taken as evidence against Darwin's notion of gradual emergence of phenomenological complexity over evolutionary time. Rather, it simply points to the view that perceptual and self-awareness likely emerged at an early stage of animal diversification in the Cambrian, or even earlier [1,6,11]. A more comprehensive study certainly requires inclusion of more than a token number of invertebrates, and more than just one or two representatives of each vertebrate class.

Zoos and aquaria provide the advantage of making observations on a number of different animals from different clades under similar conditions observable in an efficient manner. But the confinement of the animals renders their behavior divergent to lesser or greater degrees from what it might be in their natural, unconstrained habitats. The aquatic species were confined to tanks large enough for free mobility, though movements of the shark and lionfish undoubtedly were more constrained than in their natural habitats, possibly accounting for the smaller ranges in their quantitative data. Water volumes in which the lobster, cuttlefish, seahorse, and newt were kept were proportionately smaller in relation to the smaller size of those animals, which nonetheless were able to show the full range of behaviors encompassed by the criteria in the study design. All the terrestrial species were housed in spacious naturalistic settings which enabled them ample opportunity for roaming about, interacting, or being solitary. The chickens were observed in a completely open range. The importance of ethological studies in natural environments for assessing animal cognition has often been pointed out [29,47,49,50,52,53]. Nonetheless, the behaviors observed in this study, in my judgment as an experienced student of animal behavior in field environments, is generalizable to unrestricted habitats for all species with the possible exception of the shark and lionfish. Overall, this study illustrates that sequestered environments do not preclude assessment of differences in behavioral profiles across a broad range of animals.

The heat diagrams in Fig. 7 are offered as metaphorical representations of the nature of phenomenological experience for any species. They suggest a way of thinking about differences in the complexity of animal awareness, but should not be taken as literal expressions of the nature of that awareness. While, for example, the diagram for the cuttlefish resembles that of the giraffe, it should not be inferred that the mental life of the cuttlefish is necessarily like that of the giraffe. What the figures do suggest is that relatively complex phenomenological experience may exist over large phylogenetic distances.

A potential criticism of this study is that the definitions for the three modes of behavior are imprecise and call for subjective judgments by the observer. Descriptions of behavior general enough to cover species over a large range of the phylogenetic spectrum are necessarily less precise than when a single species is under investigation. However, that doesn't compromise the value of the data reported here in the context of the study's objectives for three reasons. First, all characterization of displayed behavioral modes was made by a single experienced observer whose subjective judgments and biases (to the extent present) were consistent across all observations, as supported by a CV < 40% for nine of the 12 subject species. Secondly, the many statistically significant differences between individual species and groups of species indicate that reliable species-specific differences were being recorded. Thirdly, the focus of the study was on the *pattern* of the qualitative x quantitative mosaic of phenomenological experience across species, however that experience is operationally defined. That is, the specific boundaries of each mode (as defined, even subjectively, by the observer) are less critical than the quantitative features of that mode in relation to the quantitative measures for other modes.

The behavioral modes and metrics studied here are not submitted as either criteria for phenomenology or the only aspects of experience that could point to the nature of animal minds. Grooming, object manipulation, vocalization or other forms of signaling (e.g., color changes or displays), territorial defense or aggression, gaze direction or duration, or courtship behavior could all serve as indicators for different forms of phenomenological experience. The importance of associative learning, considered by some an important indicator of cognitive complexity [11,54,55], is not denied but is not evaluated in this study.

Finally, behavior alone is not coextensive with nor a sufficient basis for assessing phenomenology. Other neuroanatomical considerations, like specific neuronal circuitry [56], brain size and number of brain cells [[57], [58], [59]], as well as CNS regions that integrate information [2,56,[60], [61], [62]], function in memory encoding and recall [11,54], and mediate states of awareness [60], are relevant to assessing the capacity for subjective experience [3]. Ultimately, neurophysiological activity must also be mapped onto states of consciousness, as it is beginning to be in humans [25,[63], [64], [65], [66]]. Cabanac et al. [67] urged the use of a roster of behavioral (play and taste aversion), psychological (pleasure in decision making and emotional tachycardia), and physiological (dopamine production, sleep, and fever) indicators for the emergence of emotion, and more broadly consciousness, during evolution. While their conclusion that consciousness first arose among early reptiles is more conservative than the implications of the results of this study, their strategy points to the importance of appreciating the broad-based neurobiological basis of phenomenological experience. Anatomy, physiology, and neurochemistry, as well as behavior and ecology, will be necessary for a full account of the nature of subjective experience in all animals capable of it [19,25].

## 5 Conclusion

To the extent that the behavioral profiles observed in this study reflect the nature of the animal's phenomenological experience, they suggest that the subjective experience of animals is heterogeneous and multimodal across species, and more closely correlates with lifestyle than with habitat or linear gradations in phylogeny. They further provide no evidence for a sharp evolutionary boundary between the absence and onset of phenomenological experience in animals.

## Funding

This research did not receive any specific grant from funding agencies in the public, commercial, or not-for-profit sectors.

## CRediT authorship contribution statement

**Louis N. Irwin:** Writing – review & editing, Writing – original draft, Methodology, Investigation, Formal analysis, Data curation, Conceptualization.

## Declaration of competing interest

The authors declare that they have no known competing financial interests or personal relationships that could have appeared to influence the work reported in this paper.

## Figures and Tables

**Table:** 

Species compared	Chi Square	df	P
Lobster v Cuttlefish	24.0	8	0.0023
Lobster v Shark	96.9	8	<0.0001
Lobster v Lionfish	66.0	8	<0.0001
Lobster v Seahorse	65.1	8	<0.0001
Lobster v Newt	52.2	8	<0.0001
Lobster v Toad*	3.61	8	invalid
Lobster v Iguana	33.0	8	<0.0001
Lobster v Chicken	122.2	8	<0.0001
Lobster v Elephant	68.3	8	<0.0001
Lobster v Giraffe	46.6	8	<0.0001
Lobster v Colobus	32.2	8	<0.0001
Cuttlefish v Iguana	51.6	8	<0.0001
Shark v Newt*	12.2	8	invalid
Shark v Iguana*	36.4	8	invalid
Lionfish v Newt*	17.4	8	invalid
Newt v Iguana*	40.5	8	Invalid
Elephant v Iguana	115.4	8	<0.0001
Elephant v Chicken	28.2	8	0.0004
Giraffe v Chicken	71.8	8	<0.0001
Monkey v. Giraffe	167.9	8	<0.0001
Monkey v. Elephant	103.0	8	<0.0001
Monkey v. Chicken	155.9	8	<0.0001
Monkey v. Iguana	105.7	8	<0.0001
Monkey v. Newt	89.6	8	<0.0001
Monkey v. Seahorse	94.97	8	<0.0001
Monkey v. Shark	121.2	8	<0.0001
Monkey v. Cuttlefish	64.7	8	<0.0001
Monkey v. Lobster	33.1	8	<0.0001
Cuttlefish v. Giraffe	57.38	8	<0.0001
Newt v. Seahorse*	82.95	8	invalid

*Analysis of data that include any values of zero is invalid in the Chi Square test.
