# Supplementary material for: Behavioral indicators of heterogeneous subjective experience in animals across the phylogenetic spectrum: Implications for comparative animal phenomenology
Source: Heliyon. 2024 Mar 24;10(7):e28421. doi: 10.1016/j.heliyon.2024.e28421 (PMC11016586; doi:10.1016/j.heliyon.2024.e28421)
Supplement: Multimedia component 1 [file mmc1.pptx]

## Slide 1
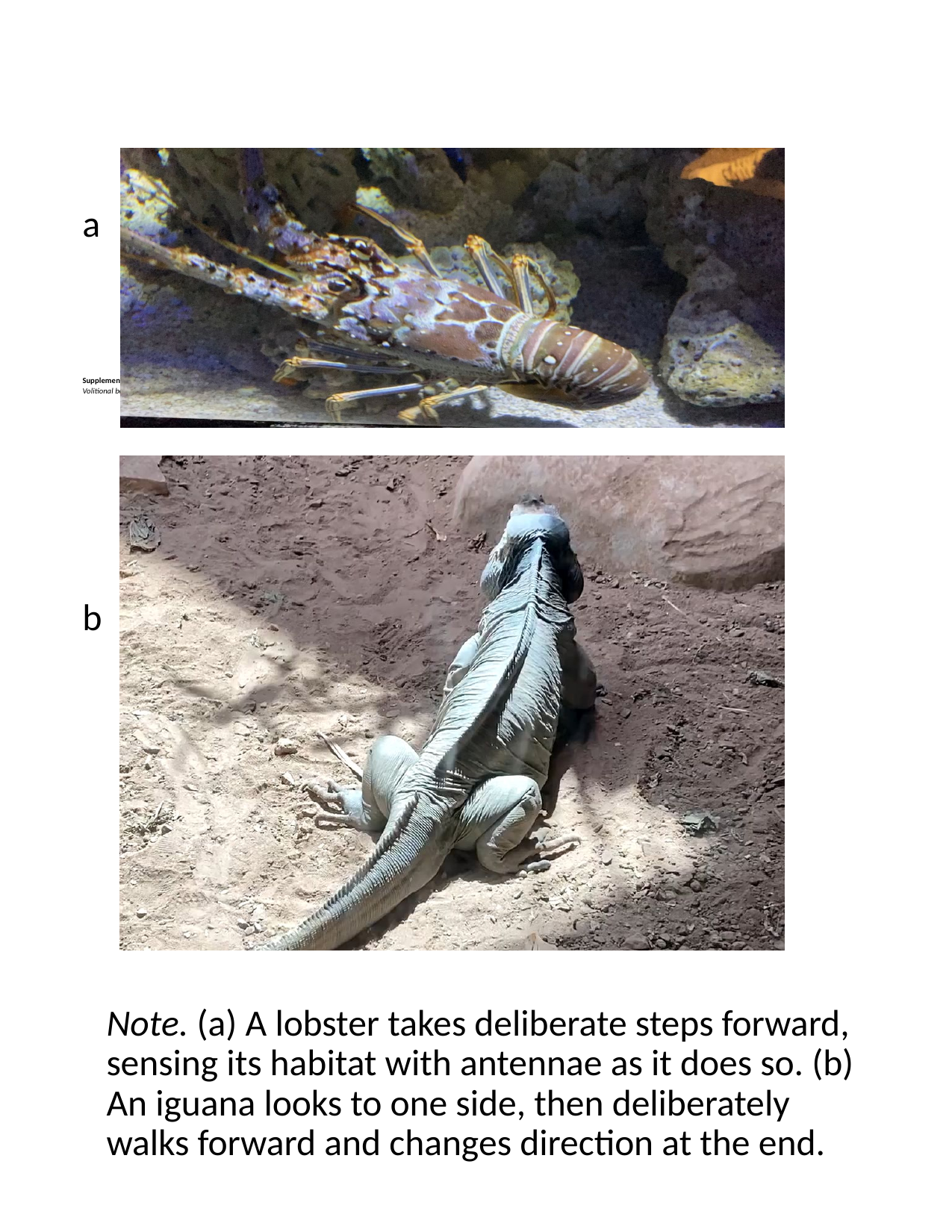

a
# Supplemental Video 1 Volitional behavior in a Lobster and Iguana
b
Note. (a) A lobster takes deliberate steps forward, sensing its habitat with antennae as it does so. (b) An iguana looks to one side, then deliberately walks forward and changes direction at the end.
