# Supplementary material for: Behavioral indicators of heterogeneous subjective experience in animals across the phylogenetic spectrum: Implications for comparative animal phenomenology
Source: Heliyon. 2024 Mar 24;10(7):e28421. doi: 10.1016/j.heliyon.2024.e28421 (PMC11016586; doi:10.1016/j.heliyon.2024.e28421)
Supplement: Multimedia component 2 [file mmc2.pptx]

## Slide 1
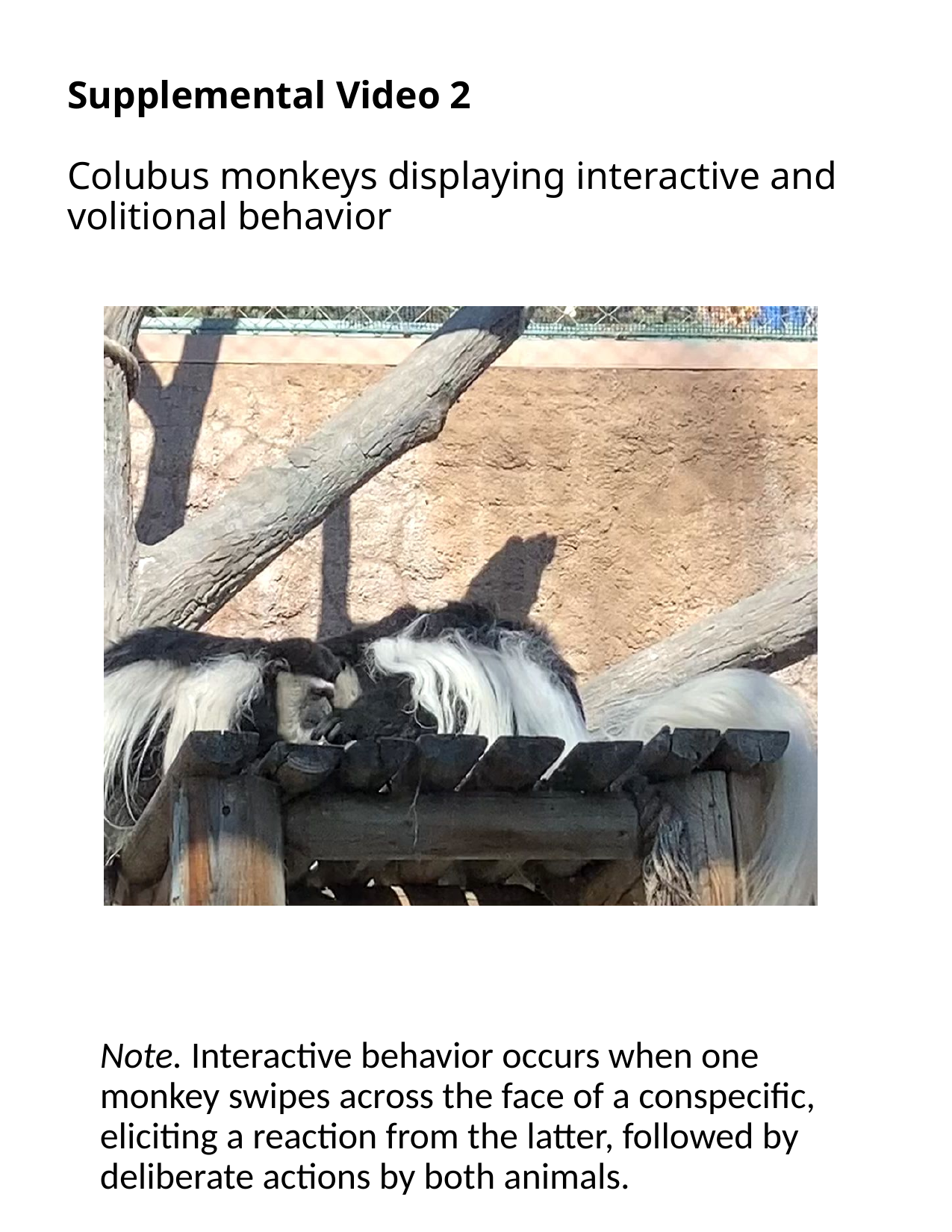

# Supplemental Video 2 Colubus monkeys displaying interactive and volitional behavior
Note. Interactive behavior occurs when one monkey swipes across the face of a conspecific, eliciting a reaction from the latter, followed by deliberate actions by both animals.
