# Supplementary material for: Behavioral indicators of heterogeneous subjective experience in animals across the phylogenetic spectrum: Implications for comparative animal phenomenology
Source: Heliyon. 2024 Mar 24;10(7):e28421. doi: 10.1016/j.heliyon.2024.e28421 (PMC11016586; doi:10.1016/j.heliyon.2024.e28421)
Supplement: Multimedia component 3 [file mmc3.pptx]

## Slide 1
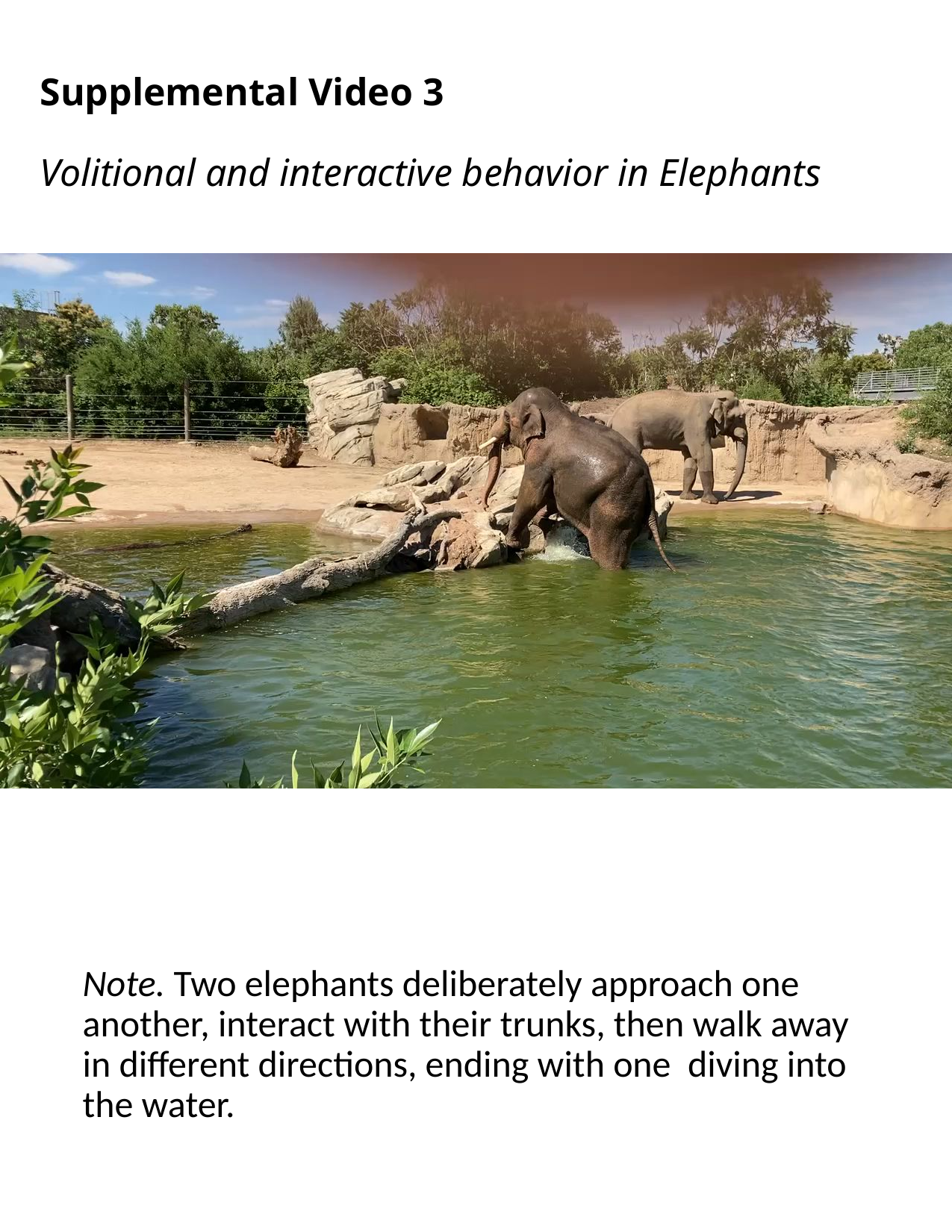

# Supplemental Video 3 Volitional and interactive behavior in Elephants
Note. Two elephants deliberately approach one another, interact with their trunks, then walk away in different directions, ending with one diving into the water.
