# Supplementary material for: Behavioral indicators of heterogeneous subjective experience in animals across the phylogenetic spectrum: Implications for comparative animal phenomenology
Source: Heliyon. 2024 Mar 24;10(7):e28421. doi: 10.1016/j.heliyon.2024.e28421 (PMC11016586; doi:10.1016/j.heliyon.2024.e28421)
Supplement: Multimedia component 4 [file mmc4.pptx]

## Slide 1
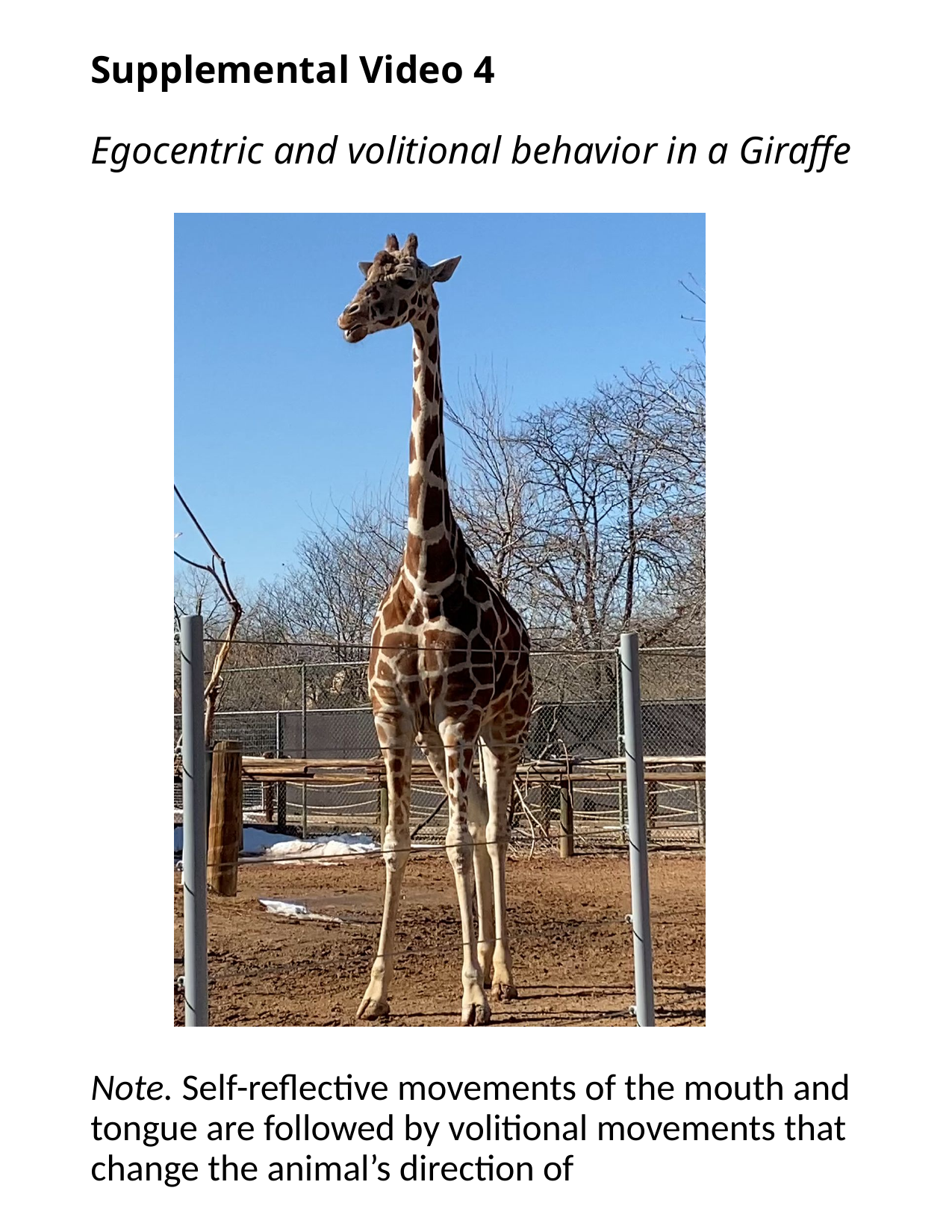

# Supplemental Video 4 Egocentric and volitional behavior in a Giraffe
Note. Self-reflective movements of the mouth and tongue are followed by volitional movements that change the animal’s direction of
